# Ultrasonographic Evaluation of Bowel Wall Thickness and Intramural Blood Flow in Ulcerative Colitis

**DOI:** 10.5402/2012/370495

**Published:** 2012-05-09

**Authors:** Abolhassan Shakeri Bavil, Mohommad Hossein Somi, Masoud Nemati, Batool Seyfi Nadergoli, Kamyar Ghabili, Reshad Mirnour, Hamideh Ashrafi

**Affiliations:** ^1^Tuberculosis and Lung Disease Research Center, Tabriz University of Medical Sciences, Tabriz 51656-65811, Iran; ^2^Liver and Gastroenterology Diseases Research Center, Tabriz University of Medical Sciences, Tabriz 51656-65811, Iran; ^3^Department of Radiology, Tabriz University of Medical Sciences, Tabriz 51656-65811, Iran; ^4^Medical Philosophy and History Research Center, Tabriz University of Medical Sciences, Tabriz 51656-65811, Iran; ^5^Students Research Committee, Tabriz University of Medical Sciences, Tabriz 51656-65811, Iran

## Abstract

*Aim*. This study aimed at assessing Doppler ultrasonographic findings of gut wall vessels and thickness in active and quiescent ulcerative colitis. *Methods*. Fifty patients with ulcerative colitis were studied using transabdominal grayscale and Doppler sonography of sigmoid, distal and middle parts of descending colon in different stages of the disease. Thickness of colon wall in the most involved site, number of color signals in each box, resistive index (RI), and pulsatility index (PI) were evaluated. *Results*. The median thickness of the colon wall in the most involved sites was 4.3 mm in acute phase and 4.4 mm in the inactive phase (*P* = 0.47). The median number of the color signals in the active phase at the most involved site, distal part of descending colon and sigmoid was higher than that of the color signals in the inactive phase (*P* = 0.0001). In the most involved site, the PI and RI were undetectable in the inactive phase. The median PI was 1.4 in the mild phase, 1.3 in the moderate phase, and 1.1 in the severe phase (*P* = 0.002). *Conclusion*. In contrast to the colon wall thickness, increased intramural blood flow reflected the clinical severity in ulcerative colitis patients.

## 1. Introduction

 Inflammatory bowel diseases, generally including ulcerative colitis and Crohn's disease, are type of intestinal diseases with still unknown etiology [1]. Ulcerative colitis, except for the severe types, merely affects the mucosa and submucosa of colon as an inflammatory ulcer. As the mucosa is edematous and its circulation may increase, studies have shown that bowel wall thickness and its circulation are in correlation with severity of the disease [2]. The congestion and dilation of capillaries in the affected area can give explanation for the increased circulation [2–4].

Transabdominal ultrasound is useful for the detection of bowel wall thickening and for determining the extent of involved segments in different kinds of inflammatory bowel diseases. Moreover, determining complications and disease activity and thereby guiding therapy decisions are achievable through ultrasonography [5–8]. However, the role of transabdominal ultrasound in ulcerative colitis has been considered less important than in Crohn's disease [7]. This conclusion is based on the conflicting data on the correlation between intramural blood flow, bowel wall thickness, and clinical severity of ulcerative colitis [7, 8]. Therefore, the aim of the present study was to assess Doppler ultrasonographic findings of gut wall vessels and bowel wall thickness in active and quiescent ulcerative colitis.

## 2. Materials and Methods

 Fifty patients diagnosed with ulcerative colitis through endoscopic evaluation along with biopsy were studied between April 2009 and July 2010. The study was approved by the local ethics committee and written informed consent was obtained for each subject. All the studied patients were on sulfasalazine plus mesalamine treatment. Transabdominal Doppler sonographic findings were assessed in different stages of the disease. Following an eight-hour fasting, transabdominal grayscale and Doppler sonography of sigmoid, distal, and middle parts of descending colon was performed by a radiologist who was unaware of the disease severity. Color Doppler box was determined as the size of the sigmoid lumen in axial section. The coronal and sagital sections were evaluated using the same color box. The size of the color Doppler box was adjusted to make it as small as possible and thus maximize sensitivity and minimize flash artifacts. Thickness of colon wall in the most involved site, number of color signals in each box, resistive index (RI), and pulsatility index (PI) were evaluated. RI is defined as (peak systolic velocity − end diastolic velocity)/peak systolic velocity. Moreover, PI is defined as (peak systolic velocity − end diastolic velocity)/mean velocity [9–11]. Clinical scoring of ulcerative colitis was based on the Truelove and Witts' classification [12].

 Data were presented as mean ± standard deviation (SD). Statistical analysis was performed with SPSS for Windows version 15.0 (Chicago, IL) using one-way ANOVA, Mann-Whitney *U* test, chi-square Test, or Fisher's Exact test, wherever appropriate. Statistical analyses were performed using computer software SPSS 15. A *P* value less than 0.05 was considered to be statistically significant.

## 3. Results

 Fifty patients with ulcerative colitis, 23 males and 27 females with mean age of  29.20 ± 8.01 years, were recruited. Left colon was the most common involved site. In terms of the disease activity index, 13 patients (26%) were in inactive phase, 12 patients in (24%) mild, 13 patients in (26%) moderate, and 12 patients (24%) in severe clinical phases. The median thickness of the colon wall in the most involved sites was 4.3 mm in acute phase and 4.4 mm in the inactive phase (*P* = 0.47). The median number of the color signals in the active phase at the most involved site, distal part of descending colon, and sigmoid was higher than that of the color signals in the inactive phase (*P* = 0.0001). In the most involved site, the PI and RI were undetectable in the inactive phase. The median PI was 1.4 in the mild phase, 1.3 in the moderate phase, and 1.1 in the severe phase (*P* = 0.002). Demographic data and ultrasonographic indices with regard to the disease severity are shown in Table 1.

## 4. Discussion

 The present study revealed that PI measured in the fasting state was significantly lower in the gut wall vessels of ulcerative colitis patients with active disease than that obtained in the inactive patients. On the other hand, the results showed that more severe cases had lower PI values in the bowel wall vessels. Furthermore, estimation of macroscopic vessel density in diseased bowel loops, based on the number of color signals, showed that patients with active disease had higher vessel density, whereas those with quiescent disease had no vessel density. This finding was significant in the most involved site, distal part of the descending colon, and sigmoid but not in the middle part of the descending colon. In addition, the more severe the disease was, the higher number of the color signals was detected. Altogether, these findings indicated that increased intramural flow reflected the clinical activity in patients with ulcerative colitis. Moreover, these results are parallel to the characteristic of the ulcerative colitis, that is, hypervascularized bowel wall [13]. Similar results were obtained both in Crohn's disease and ulcerative colitis by Ruess et al. and Shirahama et al., who reported that intramural vascularity correlated with laboratory and clinical parameters of disease activity [14, 15]. On the other hand, splanchnic flow measurements in the inferior and/or superior mesenteric arteries have been shown to be closely related to clinical and endoscopic disease activity in patients with ulcerative colitis [16–20]. However, Homann et al. and Bremner et al., failed to find any correlation between endoscopic or clinical disease severity of ulcerative colitis and superior mesenteric artery PI and RI, respectively [21, 22].

 In contrast to Crohn's disease, bowel thickening in ulcerative colitis could not be correlated with clinical disease activity in some studies [7, 15]. However, Bremner et al., found significant correlation between bowel wall thickness and endoscopic severity of both Crohn's disease and ulcerative colitis at moderate/severe stages of the diseases [22]. Furthermore, Ruess et al. concluded that bowel wall thickness correlated with common laboratory and clinical parameters of the disease activity in patients with Crohn's disease and ulcerative colitis [14]. Additionally, Maconi et al., revealed that degree of the bowel wall thickness, as evaluated by ultrasonography, correlated with the clinical, biochemical, and endoscopic activity of ulcerative colitis both before and after steroid therapy [23]. In the present paper, we failed to find any significant difference in the bowel wall thickness between the active and inactive phases of ulcerative colitis, perhaps because of the small number of patients studied.

 Different factors including meal ingestion and exercise can affect measurement of the intramural blood flow with Doppler sonography [24, 25]. Taking this into account, we evaluated our patients after overnight fasting and in resting position. We also considered the color signal box as a sonographic indicator in our study. However, the number of color Doppler signals is an estimate and not a direct count of vessels present in the area examined because a single tortuous vessel may result in several color Doppler signals.

 The diagnosis of ulcerative colitis is usually based on the patient's history and typical endoscopic appearance of the mucosa and histology after exclusion of infectious agents by microscopic examination and stool cultures. As treatment is based in part upon the extent of the disease, it is useful at the initial presentation to document the extent of inflammation, which can be accomplished by combining flexible sigmoidoscopy and ultrasound, when complete colonoscopy is not possible and/or contraindicated. However, it should be indicated that none of these sonographic findings are specific and may be also seen in a number of other colonic disorders. As a consequence, the value of transabdominal ultrasound in ulcerative colitis is less well established than in Crohn's disease [7, 26].

 This study has certain limitations. No measurement of the inflammatory markers (e.g., fecal calprotectin, C-reactive protein, etc.) was performed. Further studies covering the levels of these markers and their probable correlations with the ultrasonographic findings are recommended. In addition, the patients were not subjected to any treatment in the present study. Therefore, response to therapeutic interventions has not been evaluated. Furthermore, other ultrasound modalities including sonoelastography and contrast-enhanced sonography were not applied in our study [27–29]. However, to the best of our knowledge, the present study is the first investigation to study the number of color Doppler signals in evaluation of the inflammatory bowel diseases.

 In conclusion, this study reveals that transabdominal ultrasound, thanks to its accuracy in measuring the intramural blood flow, might be applied to the patients with ulcerative colitis to differentiate the clinical phases of the disease. Further studies in larger cohorts using updated ultrasound modalities are obviously needed to establish the value of our observation and to confirm the usefulness of transabdominal ultrasound as an important adjunctive tool for the evaluation of disease activity.

## Figures and Tables

**Table 1:** Demographic data and ultrasonographic indices in different clinical stages of ulcerative colitis, median (interquartile range).

Variable	Inactive phase (*n* = 13)	Mild (*n* = 12)	Moderate (*n* = 13)	Severe (*n* = 12)	*P* value
Age (year)	23 (8)	34.5 (21.5)	27 (4.8)	29 (8.5)	0.06
Gender (male : female)	7 : 6	6 : 6	5 : 8	5 : 7	0.85
PI (most involved site)	—	1.4 (0.4)	1.3 (0.5)	1.1 (0.6)	0.002
RI (most involved site)	—	0.7 (0.1)	0.7 (0.1)	0.6 (0.1)	0.05
Wall thickness (most involved site) (mm)	4.4 (1.8)	4.2 (0.8)	4.6 (1.9)	4.7 (1.1)	0.23
Number of color signal (most involved site)	0 (0)	1.5 (1.8)	2 (0.3)	3 (0)	0.0001
Wall thickness (middle part of descending colon) (mm)	3.3 (1.6)	3.7 (1.6)	4.1 (2.3)	3.5 (1.8)	0.64
Number of color signal (middle part of descending colon)	0 (0)	0 (0.8)	0 (0.3)	0 (1.8)	0.05
Wall thickness (distal part of descending colon) (mm)	3.7 (1.7)	3.7 (1.2)	4.4 (2.2)	4.1 (1.5)	0.53
Number of color signal (distal part of descending colon)	0 (0)	0 (0.8)	1 (2)	1 (2.5)	0.0001
Wall thickness (sigmoid) (mm)	4 (2)	4.2 (0.8)	4.6 (1.9)	4.7 (1.1)	0.13
Number of color signal (sigmoid)	0 (0)	1.5 (1)	2 (0.3)	3 (0.8)	0.0001

## References

[1] Horsthuis K, Stokkers PCF, Stoker J (2008). Detection of inflammatory bowel disease: diagnostic performance of cross-sectional imaging modalities. *Abdominal Imaging*.

[2] Ambrosini R, Barchiesi A, Di Mizio V (2007). Inflammatory chronic disease of the colon: how to image. *European Journal of Radiology*.

[3] Ibrahim CB, Aroniadis OC, Brandt LJ (2010). On the role of ischemia in the pathogenesis of IBD: a review. *Inflammatory Bowel Diseases*.

[4] Deban L, Correale C, Vetrano S, Malesci A, Danese S (2008). Multiple pathogenic roles of microvasculature in inflammatory bowel disease: a jack of all trades. *American Journal of Pathology*.

[5] Parente F, Molteni M, Marino B (2010). Are colonoscopy and bowel ultrasound useful for assessing response to short-term therapy and predicting disease outcome of moderate-to-severe forms of ulcerative colitis: a prospective study. *American Journal of Gastroenterology*.

[6] Hurlstone DP, Sanders DS, Lobo AJ, McAlindon ME, Cross SS (2005). Prospective evaluation of high-frequency mini-probe ultrasound colonoscopic imaging in ulcerative colitis: a valid tool for predicting clinical severity. *European Journal of Gastroenterology and Hepatology*.

[7] Dietrich CF (2009). Significance of abdominal ultrasound in inflammatory bowel disease. *Digestive Diseases*.

[8] Di Sabatino A, Armellini E, Corazza GR (2004). Doppler sonography in the diagnosis of inflammatory bowel disease. *Digestive Diseases*.

[9] Ghabili K, Khosroshahi HT, Shakeri A, Tubbs RS, Bahluli A, Shoja MM (2009). Can Doppler ultrasonographic indices of the renal artery predict the presence of supernumerary renal arteries?. *Transplantation Proceedings*.

[10] Nemati M, Aslanabadi S, Bavil AS (2010). Diagnostic accuracy of Doppler ultrasonography in differentiation between malignant and benign cervical lymphadenopathies in pediatric age group. *Pakistan Journal of Biological Sciences*.

[11] Ansarin K, Bavil AS, Ghabili K (2010). Are Doppler ultrasonography parameters symmetric between the right and left kidney?. *International Journal of General Medicine*.

[12] Truelove SC, Witts LJ (1955). Cortisone in ulcerative colitis; final report on a therapeutic trial. *British medical journal*.

[13] Heyne R, Rickes S, Bock P, Schreiber S, Wermke W, Lochs H (2002). Non-invasive evaluation of activity in inflammatory bowel disease by power Doppler sonography. *Zeitschrift fur Gastroenterologie*.

[14] Ruess L, Nussbaum Blask AR, Bulas DI (2000). Inflammatory bowel disease in children and young adults: correlation of sonographic and clinical parameters during treatment. *American Journal of Roentgenology*.

[15] Shirahama M, Ishibashi H, Onohara S, Miyamoto Y (2003). Application of color Doppler ultrasonography to ulcerative colitis. *Journal of Medical Ultrasonics*.

[16] Maconi G, Imbesi V, Bianchi Porro G (1996). Doppler ultrasound measurement of intestinal blood flow in inflammatory bowel disease. *Scandinavian Journal of Gastroenterology*.

[17] Ludwig D, Wieners S, Brüning A (1999). Mesenteric blood flow is related to disease activity and risk of relapse in ulcerative colitis: a prospective follow up study. *Gut*.

[18] Mirk P, Palazzoni G, Gimondo P (1999). Doppler sonography of hemodynamic changes of the inferior mesenteric artery in inflammatory bowel disease: preliminary data. *American Journal of Roentgenology*.

[19] Siğirci A, Baysal T, Kutlu R, Aladağ M, Saraç K, Harputluoğlu H (2001). Doppler sonography of the inferior and superior mesenteric arteries in ulcerative colitis. *Journal of Clinical Ultrasound*.

[20] Kalantzis N, Rouvella P, Tarazis S (2002). Doppler US of superior mesenteric artery in the assessment of ulcerative colitis. A prospective study. *Hepato-Gastroenterology*.

[21] Homann N, Klarmann U, Fellermann K (2005). Mesenteric pulsatility index analysis predicts response to azathioprine in patients with Crohn’s disease. *Inflammatory Bowel Diseases*.

[22] Bremner AR, Griffiths M, Argent JD, Fairhurst JJ, Beattie RM (2006). Sonographic evaluation of inflammatory bowel disease: a prospective, blinded, comparative study. *Pediatric Radiology*.

[23] Maconi G, Ardizzone S, Parente F, Bianchi Porro G (1999). Ultrasonography in the evaluation of extension, activity, and follow-up of ulcerative colitis. *Scandinavian Journal of Gastroenterology*.

[24] Qamar MI, Read AE (1987). Effects of exercise on mesenteric blood flow in man. *Gut*.

[25] Moneta GL, Taylor DC, Helton WS, Mulholland MW, Strandness DE (1988). Duplex ultrasound measurement of postprandial intestinal blood flow: effect of meal composition. *Gastroenterology*.

[26] Strobel D, Goertz RS, Bernatik T (2011). Diagnostics in inflammatory bowel disease: ultrasound. *World Journal of Gastroenterology*.

[27] Ishikawa D, Ando T, Watanabe O (2011). Images of colonic real-time tissue sonoelastography correlate with those of colonoscopy and may predict response to therapy in patients with ulcerative colitis. *BMC Gastroenterology*.

[28] Girlich C, Schacherer D, Jung EM, Klebl F, Huber E (2012). Comparison between quantitative assessment of bowel wall vascularization by contrast-enhanced ultrasound and results of histopathological scoring in ulcerative colitis. *International Journal of Colorectal Disease*.

[29] Yamaguchi T, Yoshida S, Tanaka S (2009). Predicting the clinical response to cytapheresis in steroid-refractory or -dependent ulcerative colitis using contrast-enhanced ultrasonography. *Scandinavian Journal of Gastroenterology*.

